# Editorial: Frontiers in malaria research

**DOI:** 10.3389/fmicb.2023.1191773

**Published:** 2023-04-03

**Authors:** Ritu Gill, Rachna Hora, Mahmood M. Alam, Abhisheka Bansal, Tarun Kumar Bhatt, Ashwani Sharma

**Affiliations:** ^1^Molecular Parasitology Lab, Centre for Biotechnology, MD University, Rohtak, India; ^2^Department of Molecular Biology and Biochemistry, Guru Nanak Dev University, Amritsar, Punjab, India; ^3^School of Infection & Immunity, University Avenue, University of Glasgow, Glasgow, United Kingdom; ^4^Molecular Parasitology Lab, School of Life Sciences, Jawaharlal Nehru University, New Delhi, India; ^5^Department of Biotechnology, Central University of Rajasthan, Bandarsindri, Ajmer, India; ^6^Laboratory of Biomolecular Research, Paul Scherrer Institute, Villigen, Switzerland

**Keywords:** malaria, *Plasmodium*, antimalarials, malaria control and elimination, malaria vaccine, cytoadherence, hypnozoites, host and vector microbiota

## Introduction

Malaria is one of the most prevalent parasitic infections caused by *Plasmodium* spp. threatening millions of people in tropical and subtropical countries. The disease affected 247 million people globally and 619,000 deaths in 2021 increasing from 229 million cases and 409,000 deaths in 2019 (WHO, 2020, 2022). The resurge in malaria burden was due to change in focus and disruption of medical facilities because of the COVID-19 pandemic. Malaria is disproportionately concentrated in WHO African region sharing about 95% of the disease burden. The children under the age of 5 years are most affected accounting for ~80% of all malaria deaths in African region (WHO, 2022). The disease posed a huge socio-economic impact on our globe and its eradication has been a health priority globally. The fight against malaria targets either the vector or the pathogen and requires new and innovative ways to accelerate the pace of progress towards malaria elimination. WHO approved RTS,S- the world's first malaria vaccine, marks a milestone in malaria research though the vaccine provides partial protection (Zavala, 2022). The seasonal administration of RTS,S vaccine along with chemoprevention drugs was found to be a promising strategy to control malaria (Chandramohan et al., 2021).

**Figure 1:**
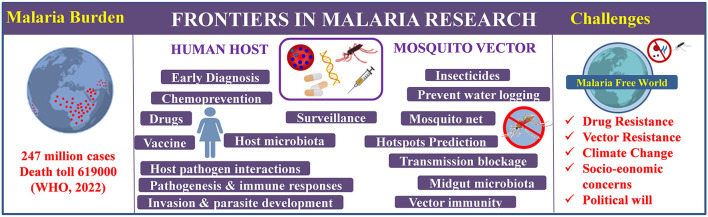
Schematic representation of global malaria burden, research frontiers and future challenges (WHO, 2022).

WHO certified twelve malaria endemic countries malaria free since 2000 and the most recent addition to the list were China and EI Salvador in 2021 highlighting the success of malaria elimination programs (WHO, 2022). However, the pace of progress toward tackling malaria is very slow in Africa- the hub of malaria burden. Further, around the globe malaria control and elimination efforts are at stake by the emergence of multidrug resistant parasite strains (Hamilton et al., 2019), insecticide resistant vectors (Suh et al., 2023) and frequently changing global climatic conditions affecting vector population and transmission dynamics rapidly (Rocklov and Dubrow, 2020; Sinka et al., 2020). Malaysia reported no indigenous case due to four human *Plasmodium* spp. for the fourth consecutive year, but malaria elimination efforts are hampered by emergence of zoonotic malaria with 3,575 *P. knowlesi* cases and 13 deaths in 2021 (WHO, 2022). A schematic representation of malaria burden, research frontiers and challenges is shown in Figure 1. To turn malaria eradication from a dream to a reality a detailed roadmap striking the right chord in a magnitude large enough to grab the pathogen is essentially required.

The Research Topic “*Frontiers in malaria research*” incorporated six publications, including three original research articles, and three reviews. This Research Topic brought forward the important findings about host and vector microbiome, parasite virulence, host parasite interactions, population genetics, thus taking one step forward in better understanding of the pathogen. The major outcomes of the studies covered are outlined below.

## Extending on Cytoadherence properties of *P. knowlesi*

Rosetting and Cytoadherence leading to sequestration of infected RBCs (IRBCs) in deep vasculature are well known phenomenon associated with severity of *P. falciparum* infections (Craig et al., 2012; Lee et al., 2019). Lee et al. demonstrated and extended the cytoadhesion properties to *P. knowlesi*-infected RBCs. *P. knowlesi* pigment-containing late stages displayed rosetting and endothelial cytoadherence phenomenon *in vitro* that was disrupted after trypsin treatment. Researchers observed differential adherence properties of IRBCs to unstimulated and stimulated human endothelial cell lines (with *P. knowlesi* culture supernatant) as well as to different types of endothelial cells. The *P. knowlesi*-IRBCs displayed low affinity to brain (hCMEC/D3) as compared to the lungs (HPMEC) and kidneys (HRGEC) endothelial cell lines. Further, the IRBCs-endothelial cytoadherence affinity increased after priming with *P. knowlesi* culture supernatant in the lungs and kidneys, however not in cerebral endothelial cells. The research work is a novel finding that provides a framework for further studies to unravel *P*. *knowlesi* ligands and host receptors involved in the phenomenon.

## Host gut microbiota and migration inhibitory factor

Another research article by Xie et al. revealed the gut microbiota changes in C57BL/6 mice after infection with *P. berghei* ANKA and in relation to the macrophage migration inhibitory factor (MIF). Human MIF is a pro-inflammatory factor and its increased level is life risk indicator in cerebral malaria (CM) (Jain et al., 2009; Rosado Jde and Rodriguez-Sosa, 2011). This work was carried out on wild type and MIF knockout mice infected with *P. berghei*. Researchers identified MIF as a regulator of host gut microbiota that further affects outcomes of CM. This study highlighted potential microbial biomarkers for *Plasmodium* infection and the mechanism through which MIF regulates CM by using a murine cerebral malaria model. The findings suggested that targeting MIF and intestinal flora together may result in effective management of severe malaria.

## Understanding hypnozoite formation in *P. vivax*

After *P. falciparum*, the second human *Plasmodium* species of concern is *P. vivax*. The parasite remains dormant in liver by transforming in hypnozoites that can cause relapses weeks, months, or years even after the parasites have been removed from the blood stream by antimalarials (Adams and Mueller, 2017). The original research by Vantaux et al. highlighted the factors that underline liver-stage fate determination (either growing schizont or hypnozoite) in *P. vivax* infections by screening different *P. vivax* patient isolates and primary human hepatocyte (PHH) lots. The findings suggested the involvement of both host hepatocytes parameters as well as *P. vivax* isolate characteristics as determinants of *P. vivax* infection progression in liver. Hepatocyte donor lot influenced hypnozoite formation rate and fating toward hypnozoites is more dependent on the *P. vivax* isolates. Further, researchers found that liver stage schizont growth was mainly influenced by the PHH donor lot. The sporozoite inoculum size was not found to be a regulator of sporozoite fating. The work underlines determinants of hypnozoite formation and need of future studies to better understand the liver stage parasite biology.

## Malaria vectors microbiome

A review by Djihinto et al. discussed the malaria-transmitting vectors microbiota as an innovative approach to target insect vectors based on “symbiotic control.” The review covered the viral, fungal, bacterial and bacteriophages diversity of *Anopheles* mosquitoes. Mosquito microbiota has been explored in search of new innovative tools to control the vector. The interplay between the vector microbiota and immune responses is one of the crucial aspects in developing mosquito resistance to the pathogen that could be exploited for effective management of the parasite (Smith et al., 2015; Wang et al., 2017). Further, authors emphasized microbe-mediated insecticide resistance mechanism in mosquitoes. Thus, a better understanding of the role of microbes in the mosquito immune system and resistance to the insecticides will help to device alternative strategies to control the malaria parasite.

## Reticulocyte tropism by malaria parasite

Erythrocyte tropism is an important phenomenon exhibited by the *Plasmodium* spp. to choose between the various developing phases of erythrocytes during their blood stage infection (Lim et al., 2017). An article by Leong et al. provided a comprehensive compilation of studies on erythrocyte tropism of ten malaria parasite species of human, non-human primates, and rodent malaria. The article highlights preference of different *Plasmodium* spp. to invade human reticulocytes and the list includes *P. vivax* (exclusively confined) *P. falciparum*, Non-human primate spp., *P. berghei* followed by *P. yoelii*. Further, a detailed glimpse of reticulocyte tropism mechanisms, advantages, drawbacks and repercussions has been discussed. The review pins reticulocytes characteristics viz. nutrient rich environment, Glucose-6-phosphate dehydrogenase elevated level, membrane stability and zoonotic potential that might be favoring parasite propagation and thus underlines the need of interventions targeting reticulocytes.

## Developments in population genetics of non-falciparum human malaria parasites

*P. falciparum*, the most deadly parasite, received all attention of researchers and policy makers to combat malaria whereas other non-falciparum *Plasmodium* spp. remain neglected. A comprehensive review by Brashear and Cui covered developments in population genomics of non-falciparum *Plasmodium* spp. vivax, malariae and ovale referred as “neglected malaria parasites”. These non-falciparum *Plasmodium* species set a challenge for malaria elimination efforts due to distinctive biological hallmarks favoring sustained transmission. Authors discussed challenges faced in whole-genome sequencing (WGS) of these non-culturable species and ways to overcome hurdles. Further, genome annotations of non falciparum malaria species need improvements and more focused inputs. Mapping SNP diversity of *P. vivax* population established differences among different populations. The authors elaborated applications of population genomics in understanding parasite biology (reticulocyte preference, invasion of Duffy-positive RBCs, relapse), observing temporal changes in parasite populations, tracking parasite origins, and migrations, identification of natural selection signatures leading parasite evolution. At the end, authors emphasized significance of development of SNP barcodes for high-throughput genotyping and need of population genomic studies in zoonotic human infecting *Plasmodium* spp. The authors presented a thought evoking review about importance of population genomics and its role in designing innovative strategies thus channelizing malaria elimination efforts.

## Conclusion and future perspectives

The contributions to the Research Topic “*Frontiers in malaria research*” focussed on revealing cytoadherence properties of *P. knowlesi*, a detailed overview of malaria-transmitting vectors microbiota, factors for liver-stage fate determination in *P. vivax*, variations in host microbiota after *Plasmodium* infection and MIF knockdown deciphering microbial biomarkers, a detailed glimpse of erythrocyte tropism of different *Plasmodium* spp. and developments in population genomics of neglected malaria parasites. The Research Topic gave thoughtful insights about basic biology and interactions of all three players of malaria- parasite, vector, and human host. The concepts gained will add on to the efforts of curtailing malaria burden. Finally, tackling the malaria parasite requires innovative tools, concerted efforts and scientific collaborations to reach malaria free world milestone.

## Author contributions

RG prepared the first draft of the manuscript. RH, MA, AB, TB, and AS revised the MS. All authors approved the final submitted version.
